# Impact of Symbiosis Between *Trichomonas vaginalis* and *Mycoplasma hominis* on Vaginal Dysbiosis: A Mini Review

**DOI:** 10.3389/fcimb.2020.00179

**Published:** 2020-05-08

**Authors:** Valentina Margarita, Pier Luigi Fiori, Paola Rappelli

**Affiliations:** ^1^Department of Biomedical Sciences, University of Sassari, Sassari, Italy; ^2^Mediterranean Center for Disease Control, Sassari, Italy

**Keywords:** bacterial vaginosis, *Mycoplasma hominis*, *Trichomonas vaginalis*, vaginal dysbiosis, symbiosis

## Abstract

The protozoon *Trichomonas vaginalis* is responsible for trichomoniasis, a common sexually transmitted infection associated with an increased risk of HIV infection and adverse pregnancy outcomes. The protozoon has the surprising ability to establish a symbiotic relationship with other microorganisms. In fact, most *T.vaginalis* isolates intracellularly host the vaginal bacterium *Mycoplasma hominis* and can harbor up to four dsRNA viruses. Moreover, a novel *Mycoplasma* species named *Ca. Mycoplasma girerdii* has been recently described as associated with trichomonad cells. *Trichomonas vaginalis* colonizes the human vagina and its presence causes profound alterations of the resident microbiota, leading to dysbiosis. In healthy women, vaginal microbiota is characterized by the presence of a complex population of aerobic and anaerobic microorganisms living in a physiologically dynamic system dominated by bacteria of the genera *Lactobacillus*. The most common microbial vaginal imbalance is bacterial vaginosis, a polymicrobial disease associated with several adverse reproductive outcomes and increased risk of HIV infection. Here, we review the current knowledge regarding the interactions between both *T.vaginalis* and *M.hominis* and the vaginal microbiota, and we discuss the possibility of a cooperation between *T.vaginalis* and its symbionts in the development of vaginal dysbiosis.

## Introduction

The protozoon *Trichomonas vaginalis* is a parasite of the human urogenital tract and is responsible for trichomoniasis, the most common non-viral sexually transmitted disease. Clinical presentation of trichomoniasis in women may vary from asymptomatic to severe vaginitis, while men tend to be mostly asymptomatic carriers of the protozoon (Petrin et al., 1998). *Trichomonas vaginalis* infection is associated with an increased risk of HIV infection and can lead to adverse pregnancy outcomes, such as preterm delivery and low birth weight. Moreover, trichomoniasis has been associated with an increased risk of cervical and prostate cancer (Stark et al., 2009).

Interestingly, the presence of the protozoon in females can profoundly alter the composition of the vaginal microbiota. The human vaginal ecosystem of women of childbearing age is characterized by the presence of a complex population of aerobic and anaerobic microorganisms that are able to establish interactions among each other and with the host in a physiologically dynamic system. Development of “next-generation” sequencing technology led to the identification of five different vaginal microbiota, which we call community state types (CSTs) (Ravel et al., 2011). Bacteria of the genus *Lactobacillus* represent the predominant microbial community in four of them (CST-I, CST-II, CST-III and CST-V), while one, CST-IV, does not show a dominant species and is composed of a more complex microbial community. Lactobacilli exert a protective effect on the host by lowering vaginal pH through lactic acid production and by competing for nutrients. Growth of adverse microbiota is also inhibited by the production of antimicrobial compounds such as bacteriocins and hydrogen peroxide.

Endogenous and exogenous factors, such as age, pregnancy, sexual intercourse and antimicrobial treatments, can alter the dynamic equilibrium of the vaginal microbiome, leading to dysbiosis of the urogenital tract. The most common microbial vaginal imbalance that occurs during reproductive age is bacterial vaginosis (BV), which affects 10–30% of women worldwide (Morris et al., 2001). Bacterial vaginosis is characterized by abnormal and malodorous vaginal discharge, an increased vaginal pH and vaginal itching. Similar to *T.vaginalis*, BV is associated with several adverse reproductive outcomes, preterm birth and premature rupture of membranes. Moreover, bacterial vaginosis also represents a risk factor for the acquisition of sexually transmitted infections (STIs) (Mirmonsef et al., 2012). Although clinical manifestations and potentially associated microbiological agents have been described in several studies, a specific etiology remains elusive, suggesting that BV is not a specific microbiological process but a polymicrobial disease caused by changes within the bacterial community (Onderdonk et al., 2016). Several studies have highlighted the main characteristics that occur during BV: a reduction of number of protective Lactobacilli and an overgrowth of anaerobic bacteria. *Gardnerella vaginalis* is the bacterium most frequently associated with BV, but other genera, such as *Atopobium, Prevotella, Bacteroides*, and *Mycoplasma*, can overgrow in case of bacterial vaginosis (Fredricks et al., 2005; Onderdonk et al., 2016). Although it is clear that several bacterial species are present during BV, the role of each single microorganism in the pathogenesis of the disease has not yet been clarified. In fact, so far, it has been impossible to identify a lone bacterial species that fits Koch's postulates for bacterial vaginosis.

Intriguingly, *T.vaginalis* can establish a symbiosis with *M.hominis*, one of the bacteria implicated in bacterial vaginosis (Rappelli et al., 1998). The presence of *M.hominis* has been demonstrated in trichomonad isolates from different geographical areas, and their strict association is the first one described so far involving two obligate human pathogens. Even though both microorganisms are able to induce disease independently in the vagina, their association has been shown to have important consequences for the pathogenicity of each of them (Dessì et al., 2019).

In this review, we focus our attention on the interplay of *Trichomonas vaginalis* and its symbiont *Mycoplasma hominis* with vaginal microbiota, and we discuss their possible role in the onset of bacterial vaginosis.

### *Trichomonas Vaginalis* and Vaginal Dysbiosis

During infection, *T.vaginalis* establishes complex interactions with the microbial community of the vagina, many of which are only partially known. An interesting study has evaluated the association between the different community state types and *T.vaginalis* and found that the 72% of women with trichomoniasis belonged to the CST-IV group (Brotman et al., 2013). This community state type is characterized by a reduced number of *Lactobacillus* species and a greater abundance of strict anaerobic bacteria, and is associated with bacterial vaginosis. Epidemiological data show that trichomoniasis is often associated with BV, but the prevalence of the two infections is probably underestimated, since both can be asymptomatic and share common features, such as the presence of vaginal discharge and inflammation (Fichorova et al., 2013). Nevertheless, the causal relationships between the two diseases are largely unknown: in other words, do particular types of vaginal bacterial communities predispose women to the acquisition of *T. vaginalis* or, on the contrary, does CST depend on the presence of the protozoon?

A longitudinal analysis showed a four- to nine-fold increased risk of trichomonad infection among sexually active women who had abnormal vaginal flora within a 3-month span, suggesting a causal role of altered microbiota on *T.vaginalis* infection (Rathod et al., 2011). *In vitro* experiments seem to support this hypothesis: in a very recent study, Hinderfeld and Simoes-Barbosa investigated the role of BV-associated bacteria on *T.vaginalis* adhesion, demonstrating that the biofilm produced by dysbiotic bacteria is able to enhance the adhesion of the protozoon to host cells, thus strengthening its pathogenic effect (Hinderfeld, 2020). Another recent study shows a cooperative interaction between *T.vaginalis* and CST-IV bacteria, resulting in an enhancement of the paracellular permeability of human ectocervical cells *in vitro* (Hinderfeld et al., 2019). Phukan et al. (2013) observed that some species of lactobacilli, and in particular *Lactobacillus gasseri*, can reduce *T.vaginalis* adhesion to vaginal cells. Since adherence to epithelial cells is a prerequisite for the cytopathic effect of the protozoon (Fiori et al., 1999), lactobacilli could exert a protective activity by reducing the pathogenicity of *T.vaginalis*, thus influencing its pathogenicity.

On the other hand, Fichorova et al. (2013) have demonstrated *in vitro* that *T. vaginalis* dramatically reduces the number of *Lactobacillus spp* associated with vaginal epithelial cells but has no effect on other species present in BV, such as *Prevotella bivia* and *Atopobium vaginae*. These findings, together with the fact that *T.vaginalis* is a phagocytic protozoon capable of ingesting and killing lactobacilli very efficiently (Juliano et al., 1991), support the hypothesis of a cause–effect relationship between trichomoniasis and bacterial vaginosis.

Another important aspect that characterizes trichomoniasis is a higher pH observed during infection than that of healthy vagina, normally ranging between 2.8 and 4.2 (McGrory et al., 1994). Since low pH is mainly due to the acidic metabolism of lactobacilli, the reduction of lactobacilli community associated with trichomoniasis leads to an increased pH, creating an environment more favorable for *T.vaginalis* growth and pathogenicity (Petrin et al., 1998). In fact, the protozoon exerts its cytopathic effect through the release of pore-forming proteins, whose activity is strictly dependent on a pH with an optimum of 5.8 (Addis et al., 1997). In this respect, the reduction of *Lactobacilli* observed during trichomoniasis could be part of a strategy aimed at the creation of an environment that best suits protozoan needs.

### *Mycoplasma hominis* and Vaginal Dysbiosis

The bacterium *Mycoplasma hominis* is an obligate parasite of the human urogenital tract belonging to the class Mollicutes and is characterized by the absence of a rigid cell wall and by one of smallest genomes among self-replicating organisms (Taylor-Robinson, 2017). *M.hominis* can colonize the human female urogenital tract of sexually mature females, and its prevalence greatly varies in studies, ranging from 1.3 to 51% (Diaz et al., 2010; Rumyantseva et al., 2019); it is influenced by several host factors, including age, sexual activity and pregnancy (Taylor-Robinson, 2017).

Although *M.hominis* can be found in both healthy and symptomatic women, several studies demonstrated that its presence is associated with vaginal flora alterations, including bacterial vaginosis and *Trichomonas vaginalis* infection. Rumyantseva et al. investigated the prevalence of *M.hominis* in 2,594 reproductive-aged women, both with healthy and altered vaginal flora, demonstrating a three-fold increase in *M.hominis* prevalence in BV patients (26.8%) compared to healthy women (8.9%) (*P* < 0.001) (Rumyantseva et al., 2019). Moreover, women with BV show a load of *M.hominis* in the vagina up to 7,500-fold higher than women without BV, suggesting that the microbiota characteristic of bacterial vaginosis may promote the growth of the bacterium. Interestingly, a synergistic relationship between *M.hominis* and the BV-associated microorganism *Gardnerella vaginalis* has been recently described. *Gardnerella vaginalis*, an anaerobic bacterium that is present in 95% of cases of BV, adheres to the vaginal epithelium and establishes a biofilm that acts as scaffold for other microorganisms contributing to the pathogenesis of the disease. Cox et al. (2016) demonstrated a higher co-infection rate of *M. hominis* and *Gardnerella vaginalis* in BV (60.7%) compared to non-BV (8.8%) women (Cox et al., 2016). Moreover, significantly higher loads of both *M.hominis* and *Gardnerella vaginalis* were detected in women with BV, suggesting a synergy between the two species. This hypothesis is corroborated by the fact that treatment of BV with metronidazole eliminates not only *G.vaginalis* and other sensitive bacteria but also *M.hominis*, that is metronidazole-resistant (Taylor-Robinson, 2017).

Other aspects characterizing vaginal dysbiosis are abundant vaginal discharge and a fishy odor, which are caused by the presence of amines (i.e., putrescine) that become malodorous in a high pH environment. The increased production of amine is caused by the great number of anaerobic bacteria that are able to produce the proteolytic enzymes that breakdown vaginal peptides (Huang et al., 2014). Recent articles showed that the presence of *M.hominis* in the vagina of women affected by BV is correlated with high levels of amine and short-chain fatty acids (SCFAs) (Vitali et al., 2015; Ceccarani et al., 2019). SCFAs include acetic, butyric and propionic acids and play an important role in a wide array of immune responses by inhibiting the production of proinflammatory cytokines, affecting immune cell migration and phagocytosis and inducing apoptosis in various cell types including neutrophils (Mirmonsef et al., 2011). These data, taken together, reinforce the hypothesis of the important role of *M.hominis* in BV condition.

### *Trichomonas vaginalis* and its Symbionts: A Microbial Cooperation Influencing Vaginal Dysbiosis?

It is well established that both *T.vaginalis* and *M.hominis*, independently, interact with members of the resident vaginal microbiota in synergistic or antagonistic ways that may influence the course of infection. Less is known about the interplay between the vaginal microbiota and the two microorganisms when they live in symbiosis.

The symbiotic relationship between *Trichomonas vaginalis* and *Mycoplasma hominis* is the first and, so far, unique case involving two obligate human parasites that are able to cause independent diseases (Dessì et al., 2019). The presence of viable *M. hominis* within *T.vaginalis* has been widely demonstrated in clinical samples from people of different geographic origin with infection rates ranging from 5% to over 89% (Fichorova et al., 2017). *Mycoplasma hominis* replicates in a coordinated fashion with trichomonad cells, and its intracellular location has been demonstrated by gentamicin protection assays and confocal and electron microscopy. In this way, *T.vaginalis* provides the bacterium with an environment protected by antibiotics activity and host immune response (Dessì et al., 2005). Moreover, *M.hominis* can be transmitted from naturally mycoplasma-infected *T.vaginalis* strains to mycoplasma-free trichomonad cells and to human epithelial cells *in vitro* (Rappelli et al., 2001), suggesting that *T.vaginalis* plays a role in transmitting the bacterium to the human host. Our group demonstrated that not only *M.hominis* but also *T.vaginalis* benefits from the symbiosis. Experiments set up on isogenic *M.hominis*-free and *M.hominis*-infected trichomonad strains demonstrated that the presence of *M.hominis* within trichomonad cells enhances the protozoan pathogenicity *in vitro*. Interestingly, the presence of *M. hominis* upregulates the secretion of proinflammatory cytokines by monocytic cells in response to *T. vaginalis*. The presence of *M.hominis* within *T.vaginalis* cells might upregulate the proinflammatory response during trichomoniasis, thus further affecting conditions associated with inflammation, such as the increased risk of acquiring cervical and prostate cancer or HIV (Dessì et al., 2019). Furthermore, *Mycoplasma* is able to influence the metabolic biochemical pathway of the protozoon, promoting a mutual benefit (Margarita et al., 2016).

The parasite pathobiology may also be influenced by the presence of RNA viruses. In fact, *T.vaginalis* can harbor up to four species of dsRNA viruses named *Trichomonas vaginalis* virus (TVV1, TVV2, TVV3, and TVV4), which have infection rates in protozoan isolates ranging from 40 to 100% (Fichorova et al., 2017). In a recent article, we showed that 51.28% of *T.vaginalis* isolates harboring *M.hominis* were infected by at least one type of TVV (Margarita et al., 2019). Interestingly, the presence of TVV can enhance (over 30-fold) the inflammatory reaction to *T.vaginalis in vitro* (Fichorova et al., 2013).

The surprising ability of *T.vaginalis* to establish symbiotic relationships with different organisms has been further confirmed by the recent discovery of a new bacterium within trichomonad cells named *Ca.M.girerdii* (Fettweis et al., 2014). This unculturable mycoplasma has been found by metagenomic studies only in women infected by *T. vaginalis*, with a vaginal microbiota characterized by a high abundance of *Prevotella* spp. and a reduced number of *Lactobacillus* species, and was associated with severe inflammation (Martin et al., 2013).

It is known that the presence of trichomonad endosymbionts may interfere with the pathogenicity of the protozoon and modify the immune response, but no data are available on its impact on the vaginal microbiota. To the best of our knowledge, no studies focusing on the effect of the consortium *T.vaginalis*/*M.hominis* on bacterial vaginosis were found, despite the findings reported above on the importance and implication of both pathogens on bacterial vaginosis. An intriguing aspect that requires further research is the role of consortium *T.vaginalis/M.hominis* on the production of vaginal biofilm during vaginal dysbiosis. The biofilm formation allows for the adhesion and successive colonization by pathogenic vaginal bacteria and confers antibiotic tolerance and resistance to host immune response. The main player in the formation of adherent biofilm on the vaginal epithelium of women with BV is *G.vaginalis* (Patterson et al., 2010), and a recent article has shown that the biofilm produced *in vitro* by *G.vaginalis* provides adhesion to the protozoan (Hinderfeld, 2020). In turn, the protozoan may provide advantages to *G. vaginalis*, since its symbiont *M. hominis* can act as a growth trigger for *G. vaginalis* during BV (Cox et al., 2016). The capability of *T.vaginalis* to establish endosymbiotic relationships with different microorganisms simultaneously (making a unique poly-microbial entity that might modify host response and parasite virulence) may have a profound impact on vaginal microbiota; this remains to be investigated.

### Preterm Birth: An Important Sequaele of *M. hominis* and *T. vaginalis* Infections and Bacterial Vaginosis

The association between vaginal infections and adverse pregnancy outcomes, such as preterm birth (PTB) and preterm prelabor rupture of membranes (PPROM), has been confirmed by several studies in the last 20 years (Guaschino et al., 2006; Lamont, 2015; Cappelletti et al., 2016). Taken together, up to 40–50 % of preterm births are associated with microbes that are able to access to the amniotic cavity and the fetus through ascension from the lower reproductive tract to the placenta, fetal membranes and uterine cavity (Fettweis et al., 2019). Both *M.hominis* and *T.vaginalis* infections are associated with complications in pregnancy. *M.hominis* is a microbe frequently isolated from both placental membranes and amniotic fluid in women with PPROM, suggesting a potential direct effect of bacteria to initiate the synthesis of prostaglandins resulting in spontaneous preterm labor (Choi et al., 2012; Capoccia et al., 2013). In contrast, *T. vaginalis* limits its infection to the vagina and is unable to reach the amniotic fluid during pregnancy, and its role in adverse pregnancy outcomes seems to be limited to the induction of a massive local inflammation with production of proinflammatory cytokines that can indirectly lead to severe pregnancy complications (Fichorova, 2009; Dessì et al., 2019). Intriguingly, our group has recently demonstrated that 58% of *M.hominis* isolated from *T.vaginalis* carry the gene *goiC* (Thi et al., 2018), which is considered a virulent trait of bacterial strains and is significantly associated with amniotic infection and preterm labor risk (Allen-Daniels et al., 2015). *Trichomonas vaginalis* could represent an additional risk factor for adverse pregnancy outcomes, as it is able to transport and protect virulent *M. hominis*, which can reach and infect the amniotic fluid. Moreover, the inflammatory response to trichomonad infection can be synergistically enhanced by the presence of the symbionts *M.hominis* and TVV.

With respect to vaginal dysbiosis, it has been shown that bacterial vaginosis acquired during the first trimester of pregnancy is associated with a five- to seven-fold increased risk of spontaneous preterm labor and PTB (Taylor-Robinson and Lamont, 2011). Although infections of individual microorganisms and resulting inflammation are considered the major risk factors for pregnancy-adverse outcomes, several critical questions remain unanswered about the role of polymicrobial infections and the interplay between pathogens and vaginal microbiota during gestation.

## Concluding Remarks

A healthy vaginal microbiota plays a major role in protecting the female genital tract against pathogenic organisms. Five different community state types have been identified based on the profile and complexity of microbiomes, and their relationships with single vaginal pathogens, including *T.vaginalis*, have been widely studied in the last years.

Nevertheless, the existence of symbiotic relations among the protozoan and the bacteria *M.hominis* and *Ca.M.girerdii* and the dsRNA viruses, leading to a complex pathogenic consortium, suggests that further studies that take into account their potential synergistic effects are necessary. In a context of vaginal dysbiosis, the microbiota, *T.vaginalis* and its endosymbionts make up a singular microbial entity that can lead to severe sequelae, including preterm delivery and the acquisition and transmission of HIV.

## Author Contributions

VM, PF and PR contributed to the conception and design of the study and wrote the manuscript. All authors contributed to manuscript revision and read and approved the submitted version.

## Conflict of Interest

The authors declare that the research was conducted in the absence of any commercial or financial relationships that could be construed as a potential conflict of interest.
